# Tips from the embryonic lung

**DOI:** 10.7554/eLife.30194

**Published:** 2017-08-14

**Authors:** Avinash Waghray, Jayaraj Rajagopal

**Affiliations:** Massachusetts General HospitalHarvard UniversityBostonUnited States

**Keywords:** lung development, branching, tip progenitor, stem cell, alveolar differentiation, organ development, Human

## Abstract

A new source of progenitor cells can now be used to study hidden aspects of human lung development and pediatric lung disease.

**Related research article** Nikolić MZ, Caritg O, Jeng Q, Johnson JA, Sun D, Howell KJ, Brady JL, Laresgoiti U, Allen G, Butler R, Zilbauer M, Giangreco A, Rawlins EL. 2017. Human embryonic lung epithelial tips are multipotent progenitors that can be expanded in vitro as long-term self-renewing organoids. *eLife* **6**:e26575. doi: 10.7554/eLife.26575

Most of our knowledge about the development of the lung comes from elegant experiments using mice (Hogan et al., 2014; Tata and Rajagopal, 2017). Despite the usefulness of mouse models, human and mouse lungs are distinct in many ways – including their size, the distribution of cell types, and the time they take to develop (Morrisey and Hogan, 2010; Wansleeben et al., 2013). Now, in eLife, Emma Rawlins and colleagues – including Marko Nikolić as first author – report that mouse and human embryonic lungs also express the same transcription factors in different patterns during development (Nikolić et al., 2017).

In both humans and mice, the lungs originate from a groove and a bud. The laryngotracheal groove becomes the future larynx and trachea, while the bud divides to become the origin of most of the branching airways and the gas-exchanging alveoli. Both bud and groove emerge from the primitive embryonic gut tube, whose epithelial layer is referred to as the embryonic anterior foregut endoderm (Figure 1A) since it originates from one of the three definitive germ layers of the embryo: the endoderm. As the embryonic lung develops, the epithelial cells at the tip of the embryonic lung proliferate most rapidly, dividing over and over to ultimately generate the beautiful branching structure of the adult organ.

**Figure 1.**
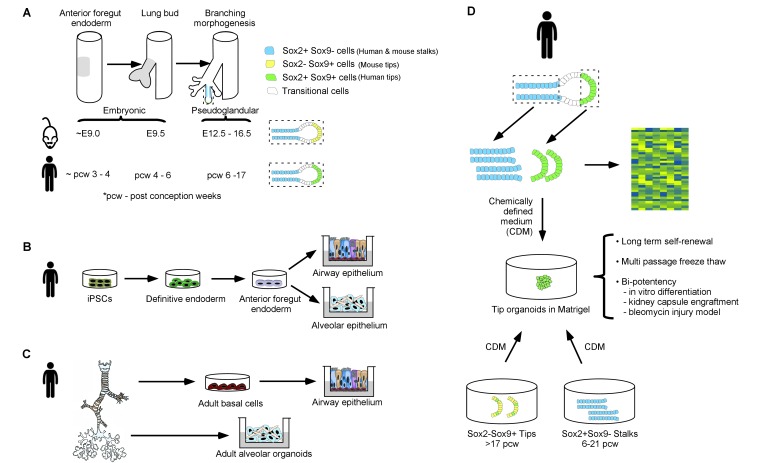
A new method to study human embryonic lung development. (**A**) The lung originates from a region of the embryonic gut, the epithelium of which is called the anterior foregut endoderm (gray shading, left tube); the numbers underneath give the age of the embryo (in days for mouse embryos and weeks for human embryos). The lung bud epithelium (gray shading, middle tube) emerges from the anterior foregut endoderm and undergoes branching morphogenesis (right tube) to give rise to stalk regions that will become the proximal airways, and a distal tip region that iteratively divides to give rise to more branching airways and eventually to alveoli (enlarged in dashed boxes). The stalk and tip regions have different expression patterns of the Sox2 and Sox9 transcription factors in humans and mice. (**B**) Induced pluripotent stem cells (iPSCs) can be differentiated in a stepwise fashion into definitive endoderm cells, then into anterior foregut endoderm and, finally, into airway and alveolar epithelial cells. (**C**) Epithelial stem cells from the airway and the alveolar compartments of the adult human lung can be manipulated in vitro to form adult airway epithelial cells (via conversion into adult basal cells) and adult alveolar organoids. (**D**) Nikolić et al. micro-dissected human lung bud tips and stalks (top) and analyzed them using global gene expression analysis (right) to identify signaling and transcriptional regulators that are expressed specifically in tip progenitor cells. This analysis was used to establish a chemically defined medium (CDM) in which human SOX2+SOX9+ cells self-renew over many passages. The SOX2+SOX9+ cells can be readily frozen and differentiated into airway and alveolar cell types for in vitro modeling and for engraftment experiments. Furthermore, the human SOX2-SOX9+/SOX2lowSOX9+ tips, characteristic of later developmental stages (greater than 17 weeks post-conception), and SOX2+SOX9 stalks (present at any stage of embryonic organ development before 21 weeks post-conception) convert into a SOX2+SOX9+ state in CDM cultures. This presumably reflects a reversion to an earlier tip progenitor state.

Many groups in the last decade have created in vitro models to study human embryonic lung development and lung disease. Early studies focused on the stepwise differentiation of induced pluripotent stem cells (iPSCs): iPSCs were first differentiated into endoderm cells and then into the lung progenitor cells that give rise to the various different epithelial cell types of the mature airway and the alveoli (Figure 1B; McCauley et al., 2017; Snoeck, 2015). Other approaches have focused on generating an expandable source of adult airway and alveolar cells directly from adult tissues (Figure 1C; Gotoh et al., 2014; Mou et al., 2016).

In humans, for obvious reasons, it has been impossible to dissect the mechanisms that underlie how lungs and other organs develop in embryos. However, we know that in mice, the transcription factor Sox2 is essential for the initiation of lung development from the gut tube, and is later expressed exclusively in the epithelial cells of the ‘stalk’ that in turn give rise to the epithelium of the airway. By contrast, tip epithelial cells express a related transcription factor, Sox9, that is necessary for the maintenance of tip cells themselves (Hogan et al., 2014; Rockich et al., 2013).

By generating an alternate and renewable source of progenitor cells from the human embryonic lung, Nikolić et al. – who are based at the University of Cambridge and University College London – now demonstrate that, unlike in the mouse, human embryonic lung tip cells produce both SOX2 and SOX9. After dissecting human embryonic tips and stalks, global gene expression analysis of the epithelium was used to identify genes specific to each cell type. By determining which growth factor-related genes were present in each of these cells, Nikolić et al. fashioned a chemically defined medium to grow tip cells as organoids. They were further able to expand, freeze and differentiate these tip-derived cells (Figure 1D).

Interestingly, the human stalk epithelial cells that produced SOX2 but did not produce SOX9 showed remarkable plasticity, and were able to give rise to “SOX2+SOX9+” tip progenitor cells. This plasticity harkens back to a classical finding from experiments performed on mice, in which tip mesenchyme cells (these are cells adjacent to the tip epithelial cells that presumably serve as a source of the growth factors that are necessary to maintain the epithelial tip cells) were grafted onto the tracheal epithelium. This grafting resulted in the formation of buds on the trachea which then started branching (Alescio and Cassini, 1962) and this phenomenon was later shown to reflect the conversion of Sox2+ tracheal epithelial cells into Sox9+ bud cells (Hogan et al., 2014). This, however, only occurred during the very early stages of lung development. Perhaps, under the right conditions, early and late stage mouse stalk cells could exhibit plasticity that mirrors the findings of Nikolić et al. in the human case, but perhaps not.

Notably, the cultured human SOX2+SOX9+ tip cells differentiate into both airway and alveolar cells when grown in vitro or in mice. Whether the differentiated cells fully mature and become fully functional remains to be seen, but the results are promising.

The hitherto unknown facets of human embryonic lung development that Nikolić et al. reveal can now be mechanistically scrutinized. The human SOX2+SOX9+ cells they cultured could potentially be used to model pediatric lung diseases (such as bronchopulmonary dysplasia), which are thought to originate during lung development. Indeed, because human SOX2+SOX9+ cells are derived from embryonic lungs, they are likely to more faithfully reflect the epigenetic features of the embryonic cells that contribute to pediatric lung disease than cells derived from adult lung epithelial cells or iPSCs. For example, iPSC-derived lung cells could possess abnormal epigenetic marks and/or the epigenetic and genetic state of cells derived from adult lungs may reflect environmental assaults encountered in the course of adulthood that are not relevant for fetal and postnatal lung disease. Thus the availability of embryonic lung epithelial cells is likely to open up whole new fields of important inquiry.

## References

[bib1] Alescio T, Cassini A (1962). Induction in vitro of tracheal buds by pulmonary mesenchyme grafted on tracheal epithelium. Journal of Experimental Zoology.

[bib2] Gotoh S, Ito I, Nagasaki T, Yamamoto Y, Konishi S, Korogi Y, Matsumoto H, Muro S, Hirai T, Funato M, Mae S, Toyoda T, Sato-Otsubo A, Ogawa S, Osafune K, Mishima M (2014). Generation of alveolar epithelial spheroids via isolated progenitor cells from human pluripotent stem cells. Stem Cell Reports.

[bib3] Hogan BL, Barkauskas CE, Chapman HA, Epstein JA, Jain R, Hsia CC, Niklason L, Calle E, Le A, Randell SH, Rock J, Snitow M, Krummel M, Stripp BR, Vu T, White ES, Whitsett JA, Morrisey EE (2014). Repair and regeneration of the respiratory system: complexity, plasticity, and mechanisms of lung stem cell function. Cell Stem Cell.

[bib4] McCauley KB, Hawkins F, Serra M, Thomas DC, Jacob A, Kotton DN (2017). Efficient derivation of functional human airway epithelium from pluripotent stem cells via temporal regulation of wnt signaling. Cell Stem Cell.

[bib5] Morrisey EE, Hogan BL (2010). Preparing for the first breath: genetic and cellular mechanisms in lung development. Developmental Cell.

[bib6] Mou H, Vinarsky V, Tata PR, Brazauskas K, Choi SH, Crooke AK, Zhang B, Solomon GM, Turner B, Bihler H, Harrington J, Lapey A, Channick C, Keyes C, Freund A, Artandi S, Mense M, Rowe S, Engelhardt JF, Hsu YC, Rajagopal J (2016). Dual SMAD signaling inhibition enables long-term expansion of diverse epithelial basal cells. Cell Stem Cell.

[bib7] Nikolić MZ, Caritg O, Jeng Q, Johnson JA, Sun D, Howell KJ, Brady JL, Laresgoiti U, Allen G, Butler R, Zilbauer M, Giangreco A, Rawlins EL (2017). Human embryonic lung epithelial tips are multipotent progenitors that can be expanded in vitro as long-term self-renewing organoids. eLife.

[bib8] Rockich BE, Hrycaj SM, Shih HP, Nagy MS, Ferguson MA, Kopp JL, Sander M, Wellik DM, Spence JR (2013). Sox9 plays multiple roles in the lung epithelium during branching morphogenesis. PNAS.

[bib9] Snoeck HW (2015). Modeling human lung development and disease using pluripotent stem cells. Development.

[bib10] Tata PR, Rajagopal J (2017). Plasticity in the lung: making and breaking cell identity. Development.

[bib11] Wansleeben C, Barkauskas CE, Rock JR, Hogan BL (2013). Stem cells of the adult lung: their development and role in homeostasis, regeneration, and disease. Wiley Interdisciplinary Reviews: Developmental Biology.

